# *Arsenophonus* GroEL Interacts with CLCuV and Is Localized in Midgut and Salivary Gland of Whitefly *B. tabaci*

**DOI:** 10.1371/journal.pone.0042168

**Published:** 2012-08-10

**Authors:** Vipin Singh Rana, Shalini Thakur Singh, Natarajan Gayatri Priya, Jitendra Kumar, Raman Rajagopal

**Affiliations:** Department of Zoology, University of Delhi, Delhi, India; Universidade Federal do Rio de Janeiro, Brazil

## Abstract

Cotton leaf curl virus (CLCuV) (*Gemininiviridae: Begomovirus*) is the causative agent of leaf curl disease in cotton plants (*Gossypium hirsutum*). CLCuV is exclusively transmitted by the whitefly species *B. tabaci* (Gennadius) (Hemiptera: Alerodidae). *B. tabaci* contains several biotypes which harbor dissimilar bacterial endo-symbiotic community. It is reported that these bacterial endosymbionts produce a 63 kDa chaperon GroEL protein which binds to geminivirus particles and protects them from rapid degradation in gut and haemolymph. In biotype B, GroEL protein of *Hamiltonella* has been shown to interact with Tomato yellow leaf curl virus (TYLCV). The present study was initiated to find out whether endosymbionts of *B. tabaci* are similarly involved in CLCuV transmission in Sriganganagar (Rajasthan), an area endemic with cotton leaf curl disease. Biotype and endosymbiont diversity of *B. tabaci* were identified using MtCO1 and 16S rDNA genes respectively. Analysis of our results indicated that the collected *B. tabaci* population belong to AsiaII genetic group and harbor the primary endosymbiont *Portiera* and the secondary endosymbiont *Arsenophonus*. The GroEL proteins of *Portiera* and *Arsenophonus* were purified and *in-vitro* interaction studies were carried out using pull down and co-immunoprecipitation assays. *In-vivo* interaction was confirmed using yeast two hybrid system. In both *in-vitro* and *in-vivo* studies, the GroEL protein of *Arsenophonus* was found to be interacting with the CLCuV coat protein. Further, we also localized the presence of *Arsenophonus* in the salivary glands and the midgut of *B. tabaci* besides the already reported bacteriocytes. These results suggest the involvement of *Arsenophonus* in the transmission of CLCuV in AsiaII genetic group of *B. tabaci*.

## Introduction

Geminiviruses are a group of plant viruses that infect a wide range of dicotyledonous crops. They cause huge losses to the world agricultural economy in the tropical and subtropical regions [1], [2], [3], [4]. *Cotton leaf curl virus* (CLCuV) is a monopartite begomovirus belonging to family geminiviridae which causes leaf curl disease in cotton plants (*Gossypium hirsutum*). CLCuV genome contains a single circular ssDNA molecule of size 2.5–3.0 kb, designated as DNA A which encodes for 6 genes (AC1, AC2, AC3, AC4, AV1 and AV2) that are essential for viral replication in the host plant. Beside DNA A, CLCuV also contains a 1.3 kb DNA molecule known as DNA β which is required for replication and encapsidation of the DNA A component, hence it is also known as satellite DNA [5].

CLCuV as well as all the other 114 species of Begomoviruses are vectored exclusively by *B. tabaci* [6]. *B. tabaci* is a sap sucking hemipteran insect belonging to family *Aleyrodidae*. It is a polyphagous insect which can survive on more than 700 species of plants in 86 families and causes severe damage to crops both, directly by feeding and indirectly by transmitting plant viruses. *B. tabaci* is a species complex consisting of 12 different genetic groups and more than 24 biotypes [7], [8] that can be distinguished by DNA markers and biological characters like dispersal, reproductive rate and host plant damaging efficiency. Since, various methodologies led to renaming and overlapping of different biotypes of *B. tabaci* Boykin *et al.*, 2007 [9], have divided the world *B. tabaci* population into 12 major well resolved genetic groups using Bayesian analysis. Like many other hemipterans, *B. tabaci* also feeds on phloem sap which, although is rich in carbohydrates, but lacks essential amino acids. These lacking nutrients are expected to be compensated by the bacterial community harbored by the insect [10]. The endosymbotic bacterial populations of *B. tabaci*, have been divided into two groups namely the obligate primary endosymbionts and the facultative secondary endosymbionts [11]. Besides *Portiera* [12], which is the primary endosymbiont, *B. tabaci* is also known to harbor many secondary endosymbionts; like *Wolbachia* [13], *Rickettsia* [14], *Cardinium* [15], *Arsenophonus* [16], *Hamiltonella* [17], and *Fritschea* [18]. In addition to providing nutrients to their insect host, these endosymbionts also confer them with functional abilities such as temperature tolerance [19] increased resistance to parasites [20], increased resistance to insecticides [21], sex determination [22], etc. The endosymbionts of *B. tabaci* as well as other insect vector hosts are reported to play a major role in virus transmission. Studies have shown that the GroEL protein of *Buchnera aphidicola*, endosymbiont of *Myzus persicae* binds to the luteovirus coat protein and protect virus particles from rapid proteolysis in gut and haemolymph [23]. Likewise in *B. tabaci*, GroEL homologue secreted by *Hamiltonella* endosymbiont has been shown to interact with TYLCV (*Tomato yellow leaf curl virus*) coat protein [24]. It has also been reported that interrupting the interaction of GroEL and coat protein leads to a dramatic decrease in the virus transmission efficiency in *B. tabaci* [25], [26]. There are also contradictory reports about the role of endosymbionts in virus transmission, for example Bouvaine *et al.*, [27] reported that GroEL protein secreted by *Buchnera aphidicola* the endosymbiont of *Myzus persicae* is found confined to the bacterial cells and is neither present in the haemolymph nor in the gut and fat body of the insect host.

India is the second largest producer of cotton and this crop contributes immensely in maintaining the high growth rate of Indian economy [28], [29]. The first outbreak of cotton leaf curl disease (CLCuD) in India was reported from Sriganganagar area (Rajasthan, India) in1993 [30]. Later in 1994, this disease had also appeared in the neighboring states of Haryana and Punjab.

In previous studies, Sriganganagar was reported to have a heavy infection of Cotton leaf curl Rajasthan virus (CLCuRV); (accession number- EF057791.1) [31]. During our survey of this region we found that cotton crop health was very poor due to CLCuV infection as well as serious infestation of *B. tabaci*. The information about indigenous *B. tabaci* population in India is poorly understood and its biotypes as well as endosymbionts are largely inscrutable. Hence, in the present study, we have identified the biotype and the native endosymbionts harbored in the *B. tabaci* population from Sriganganagar, India. Further, to find out the role of endosymbionts in begomovirus transmission, GroEL proteins of the identified endosymbionts were cloned, sequenced, heterologously expressed, purified and checked for interaction with purified CLCuV coat protein using *in-vitro* interaction studies and yeast two hybrid experiments.

## Results

### *B. tabaci* population in Sriganganagar belongs to Asia II genetic group

**Figure 1:**
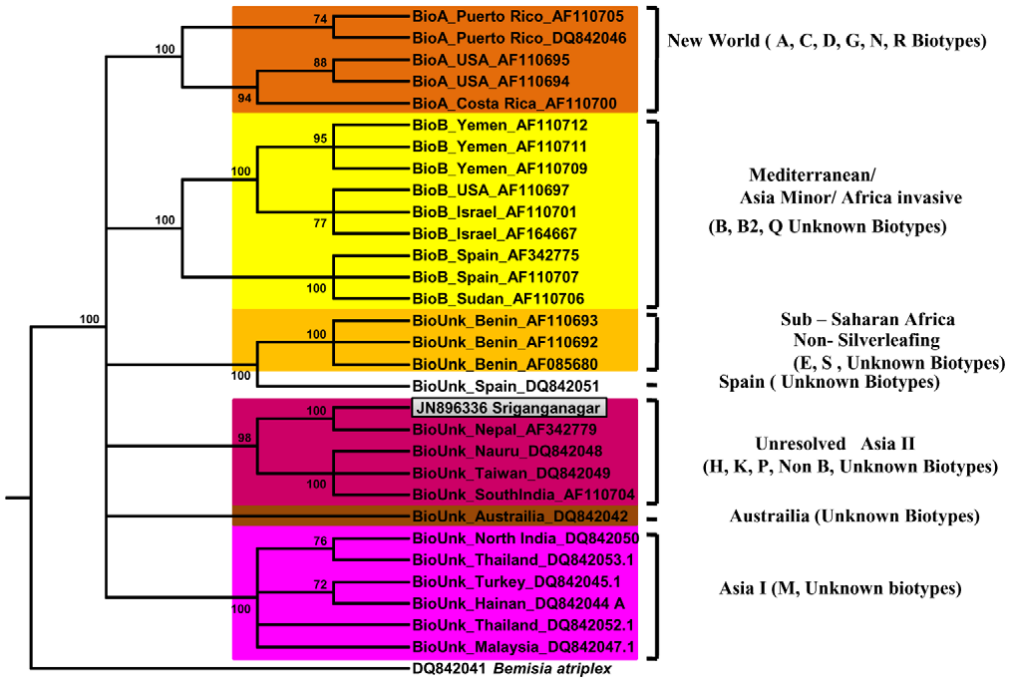
Phylogenetic analysis of *B. tabaci* Mt COI gene sequences used by Boykin *et al.* [**7**] and sequence generated from this study. Phylogenetic tree was constructed by Maximum Likelihood analysis using PHYLIP version 3.69. We used the sequences in figure 2 of Boykin *et al.* (8) and included the sequence generated in this study to identify its genetic grouping. Only bootstrap values higher than 70% are indicated. Sequence generated in this study is in grey coloured box.

The world population of *B. tabaci* has been grouped into 12 well resolved genetic groups based on Bayesian phylogenetic analysis of the *mtCO1* DNA sequences [9]. Many recent publications have used Boykin *et al., 2007* [9] as the basis of identification of their respective *B. tabaci* population [32], [24], [13] and hence we also adopted the same and constructed the phylogenetic tree using *mtCO1* gene sequences used in figure 2 of Boykin *et al., 2007* [9] along with the *mtCO1* gene sequence of *B. tabaci* from Sriganganagar. The outgroup chosen is DQ842041 (*Bemisia atriplex*) which is same as used in figure 2 of Boykin *et al.* [9] as it also belongs to the genus *Bemisia*. It was found that our whitefly populations clustered with the Asia II genetic group (Figure 1). NCBI gene bank accession number of *mtCO1* sequence generated in the present study is JN896336.

### Endosymbiont population in *B. tabaci* collected from Sriganganagar was less diversified

**Figure 2:**
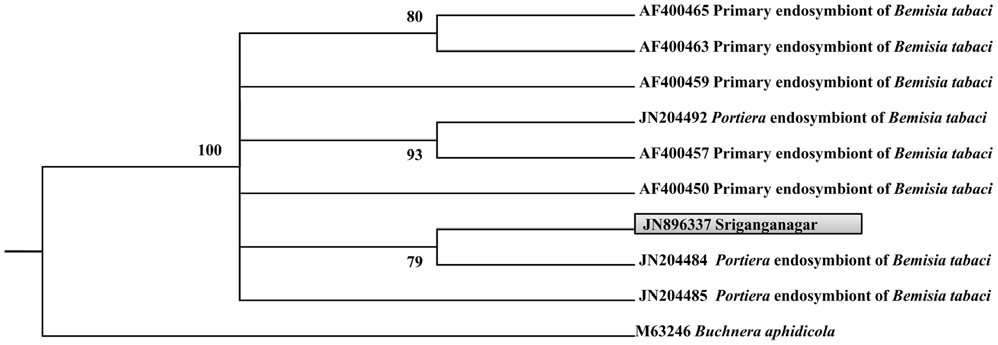
Phylogenetic analysis of 16SrDNA *Portiera* sequences obtained from NCBI database and sequence generated from this study. Phylogenetic analysis was constructed by maximum likelihood analysis using PHYLIP version 3.69. Only bootstrap values higher than 70% are indicated. Sequence generated in this study is in shaded box.

**Figure 3:**
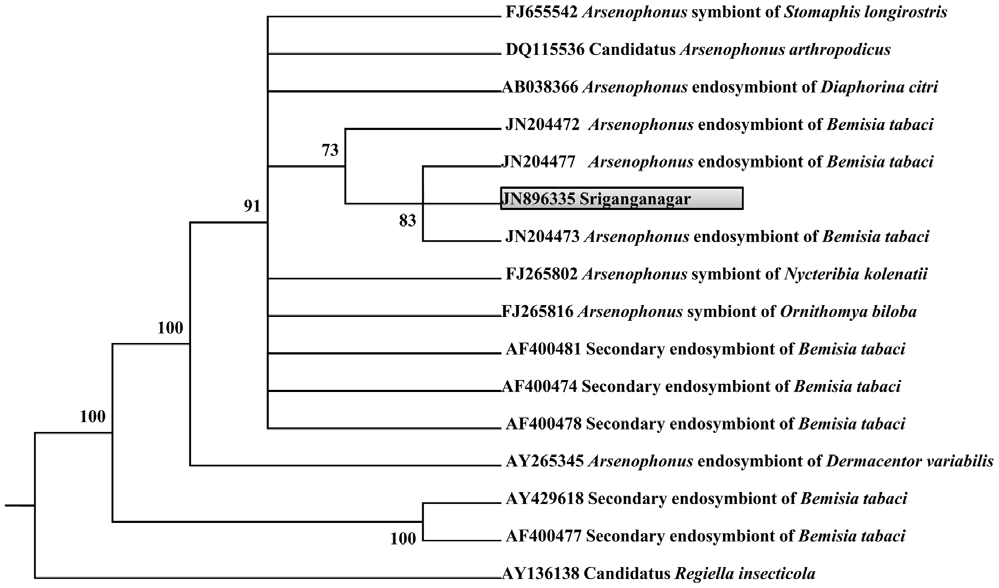
Phylogenetic analysis of 16SrDNA *Arsenophonus* sequences obtained from NCBI database and sequence generated from this study. Phylogenetic tree was constructed by maximum likelihood analysis using PHYLIP version 3.69. Only bootstrap values higher than 70% are indicated. Sequence generated in this study is in shaded box.

Analysis of the 16S rDNA sequences of 50 clones from the 16S rDNA library showed the presence of two bacteria. Phylogenetic analysis of these two sequences revealed their identity as the primary endosymbiont *Portiera* (16 clones); (Figure 2) and the secondary endosymbiont *Arsenophonus* (34 clones); (Figure 3). It was seen that *Arsenophonus* was more abundant than *Portiera* constituting 68% and 32% of the population respectively. 16S rDNA sequences of *Portiera* and *Arsenophonous* have been submitted to NCBI database under accession numbers JN896337 and JN896335.

### Isolation and Purification of CLCuV coat protein, and GroEL protein from *Portiera, Arsenophonus* and *E. coli*

Attempts to heterologously express CLCuV coat protein using pGEX4T1 in *E. coli BL-21* strain failed. Expression of the 52 kDa GST tag coat protein was achieved by using *E. coli* strain *Rossetta gami 2 DE3 PLysS* and 20% of the protein was in the soluble fraction which was further purified and used for interaction studies (Figure S1.a, b). Western blotting was performed with Anti- coat protein polyclonal antibody which confirmed the identity of the protein (Figure S1.c).

Using specific primers, *Portiera* and *E. coli* GroEL genes were amplified and cloned. A similar approach could not be adopted for cloning *Arsenophonus* GroEL, since the GenBank database has no record of *Arsenophonus* GroEL from whitefly, or from any other insect of the sub-order Homoptera. Hence, we designed primers based on the GroEL sequences of *A. nasoniae* and cloned a 1680 bp fragment. Sequence analysis of this gene indicated that it is 92% similar to *Arsenophonus nasoniae* GroEL, thus confirming that the gene belongs to *Arsenophonus* present in *B. tabaci*. Sequences of *Portiera* and *Arsenophonus* GroEL gene have been deposited under accession no. JN896338 and JN896334 respectively in NCBI database.

*Portiera, E. coli* and *Arsenophonus* GroEL genes were heterologously-expressed using *E. coli BL-21* expression strain. All the three heterologously expressed GroEL proteins were in soluble fraction (Figure S2. a, b, c) and were purified using standard protocols. *Arsenophonus* and *E. coli* GroEL proteins showed vigorous expression pattern while, *Portiera* GroEL protein showed weak expression level in *E. coli BL-21*. The identities of all the three GroEL proteins were confirmed by Western blotting with Anti-*E. coli* GroEL antibody (Figure S3.a, b).

### *Arsenophonus* GroEL protein interacts with CLCuV coat protein, while *Portiera* and *E. coli* GroEL do not interact

**Figure 4:**
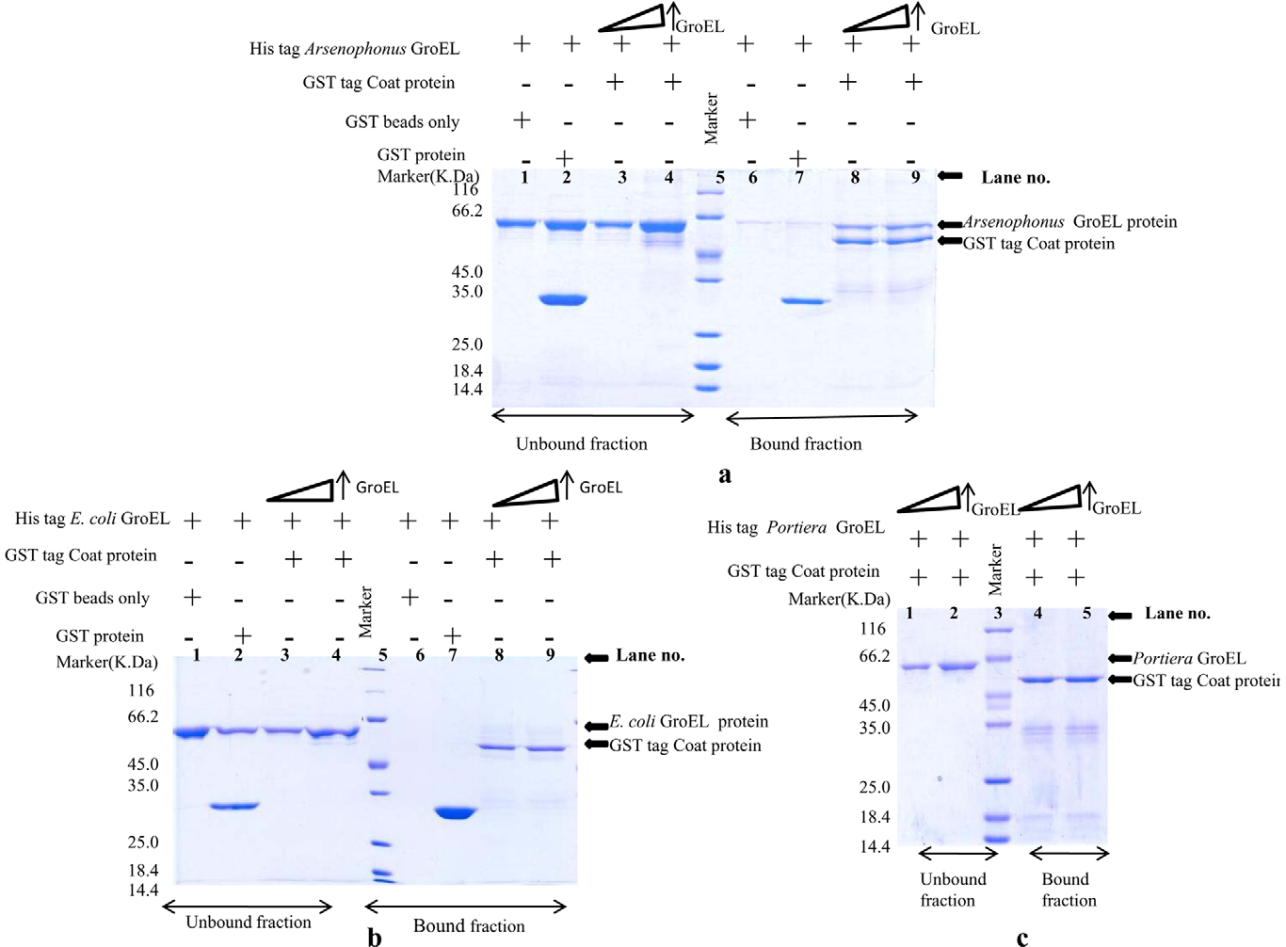
Pull down assay. Pull down assay was performed to study the interaction of CLCuV coat protein with all purified GroEL proteins. In each pull down assay, GST tagged coat protein was immobilized on glutathione-s-transferase agarose beads on which purified HIS tagged GroEL protein which interacts with the coat protein would only get immobilized. After elution, proteins were subjected to a SDS PAGE and stained with coomassie brilliant blue dye. **Lane 5-** Molecular weight marker. **Figure 4.a.** ***In-vitro*** **interaction of HIS tagged** ***Arsenophonus*** **GroEL protein with GST tagged coat protein.** **Lane 5-** Molecular weight marker **Lanes 1–4** are unbound fractions and the corresponding bound fractions are shown in **lane 6–9**. As a control, HIS tagged *Arsenophonus* GroEL of size ∼66 kDa was incubated with GST beads alone and purified GST protein of size 28 kDa as shown in **lanes 1 and 2** and their respective bead elutes are shown in **lanes 6 and 7**. **Lanes 6 and 7-** no GroEL band was observed indicating *Arsenophonus* GroEL is not interacting with GST beads and GST protein. **Lanes 3, 4**- unbound fractions of HIS tag *Arsenophonus* GroEL and GSTtagged CLCuV coat protein. No band of GST tagged CLCuV coat protein was observed in the unbound fraction indicating that *Arsenophonus* GroEL is bound to the coat protein. **Lanes 8 and 9-** corresponding bound fractions of HIS tag *Arsenophonus* GroEL and GST tagged CLCuV coat protein. A 52 kDa band of GST tag CLCuV coat protein was observed along with the ∼66 kDa band of HIS tagged *Arsenophonus* GroEL protein implying that GroEL protein of *Arsenophonus* is interacting with CLCuV coat protein. **Lane 4 and 9-** contained double the amount of *Arsenophonus* GroEL protein than lane 3 and 8 respectively. No significant change in interacting bands was seen with increased GroEL protein. The key at the top of the figure indicates the various combinations of HIS tagged *Arsenophonus* GroEL, GST tagged coat protein, GST beads only and GST protein. (+) indicates presence of the constituents while (−) indicates absence of the constituents in the respective lane. **Figure 4.b.** ***In-vitro*** **interaction of HIS tagged** ***E. coli*** **GroEL protein with GST tagged CLCuV coat protein.** **Lane 5-** Molecular weight marker. **Lanes 1–4** are unbound fractions and the corresponding bound fractions are shown in **lane 6–9**. As a control, HIS tagged *E. coli* GroEL of size ∼66 kDa was incubated with GST beads alone and purified GST protein of size 28 kDa as shown in **lanes 1 and 2** and their respective bead elutes are shown in **lanes 6 and 7. Lanes 6 and 7-** no GroEL band was observed indicating *E. coli* GroEL is not interacting with GST beads and GST protein. **Lanes 3, 4**- unbound fractions of HIS tag *E. coli* GroEL and GSTtagged CLCuV coat protein. No band of GST tagged CLCuV coat protein was observed in the unbound fraction indicating that *E. coli* GroEL is bound to the coat protein. **Lanes 8 and 9-** corresponding bound fractions of HIS tag *E. coli* GroEL and GST tagged CLCuV coat protein. A 52 kDa band of GST tag CLCuV coat protein was observed along with the ∼66 kDa band of HIS tagged *E. coli* GroEL protein implying that GroEL protein of *E. coli* is interacting with CLCuV coat protein. Lane 4 and 9 contained double the amount of *E. coli* GroEL protein than lane 3 and 8 respectively. No significant change in interacting bands was seen with increased GroEL protein. The key at the top of the figure indicates the various combinations of HIS tagged *E. coli* GroEL, GST tagged coat protein, GST beads only and GST protein. (+) indicates presence of the constituents while (−) indicates absence of the constituents in the respective lane. **Figure 4.c.** ***In-vitro*** **interaction of HIS tagged** ***Portiera*** **GroEL and GST tagged CLCuV coat protein. Lanes 1 and 2-** are unbound fractions and corresponding bound fraction are shown in **lanes 4 and 5. Lane 3-** molecular weight marker. **Lanes 1, 2 and 4, 5-** no ∼66 kDa band of *Portiera* GroEL was seen, no interaction was observed in bound fractions. Lanes 2 and 5 contain twice the amount of *Portiera* GroEL than lanes 1 and 4. The key at the top of the figure indicates the various combinations of HIS tagged *Portiera* GroEL, GST tagged coat protein, GST beads only and GST protein. (+) indicates presence of the constituents while (−) indicates absence of the constituents in the respective lane.

Interaction studies were performed using pull down, immunoprecipitation and yeast two hybrid assays. Result of pull down assays suggested a strong interaction between CLCuV coat protein and *Arsenophonus* GroEL (Figure 4.a). *E. coli* and *Portiera* GroEL protein did not show interaction with CLCuV coat protein in pull down assay (Figure 4.b, c); (It must be mentioned that some very weak interaction was observed with *E. coli* GroEL and it did not sustain on rigorous washing with high concentration of salt). It was further noticed that *Arsenophonus* GroEL and coat protein complex was stable even when washed with buffer containing 700 mM NaCl and 1% NP40 (Data not shown).

**Figure 5:**
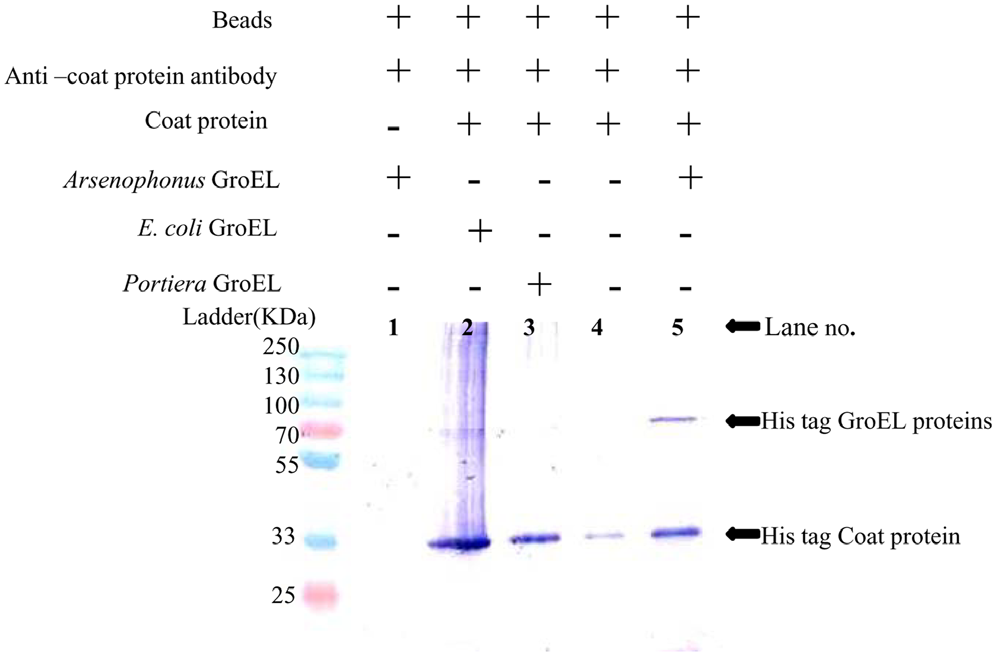
The co- immunprecipitation of HIS tagged CLCuV coat protein with HIS tagged GroEL proteins of *Arsenophonus*, *Portiera* and *E. coli*. Individual HIS tagged GroEL proteins of *Arsenophonus*, *Portiera* and *E. coli* were coexpressed with HIS tagged CLCuV coat protein in *Rossetta gami 2 DE3 PlysS*. The coexpressed *Rossetta gami 2 DE3 PlysS* cell lysate was incubated with resin bound with anti-coat protein antibody (raised in rabbit). **Lane 1-** As a control *Arsenophonus* GroEL lysate was incubated with resin and anti-coat protein antibodies. No non specific band was seen hence ruling out any non specific interaction. **Lane 2-** a very faint band of *E.coli* GroEL(∼66 kDa) was seen, indicating the weak interaction of *E. coli* GroEL protein with CLCuV coat protein. **Lane 3-** no band of *Portiera* GroEL protein was observed indicating that *Portiera* GroEL protein is not interacting with CLCuV coat protein. **Lane 4-** As a control CLCuV coat protein alone was also incubated with resin and anti- coat protein antibodies. A 30 kDa band was seen showing that CLCuV coat protein was binding to anti-coat protein antibodies. **Lane 5-** A ∼66 kDa band of HIS tagged *Arsenophonus* GroEL was seen along with a 30 kDa HIS tagged CLCuV coat protein band implying there is a specific interaction between *Arsenophonus* GroEL protein and CLCuV coat protein.

To perform immuno-precipitation, genes were coexpressed in appropriate combinations like *Arsenophonus* GroEL- CLCuV Coat protein, *Portiera* GroEL - CLCuV Coat protein, *E. coli* GroEL - CLCuV Coat protein. Analysis of the results suggested that *Arsenophonus* GroEL interacts with CLCuV coat protein while, *Portiera* GroEL did not show any interaction (Figure 5). As in the pull down assay, *E. coli* GroEL showed weak interaction with CLCuV coat protein. To check the specificity of interaction of *Arsenophonus* GroEL with CLCuV coat protein, the lysates expressing *Arsenophonus* and CLCuV coat protein each were separately incubated with Anti- Coat protein antibody and resin. Result of these controls showed that coat protein interacts with anti-coat protein antibody and resin whereas, *Arsenophonus* GroEL alone did not show interaction with anti-coat protein antibody and resin, which further suggested that no non specific binding was involved in the interaction of *Arsenophonus* GroEL with CLCuV coat protein.

**Figure 6:**
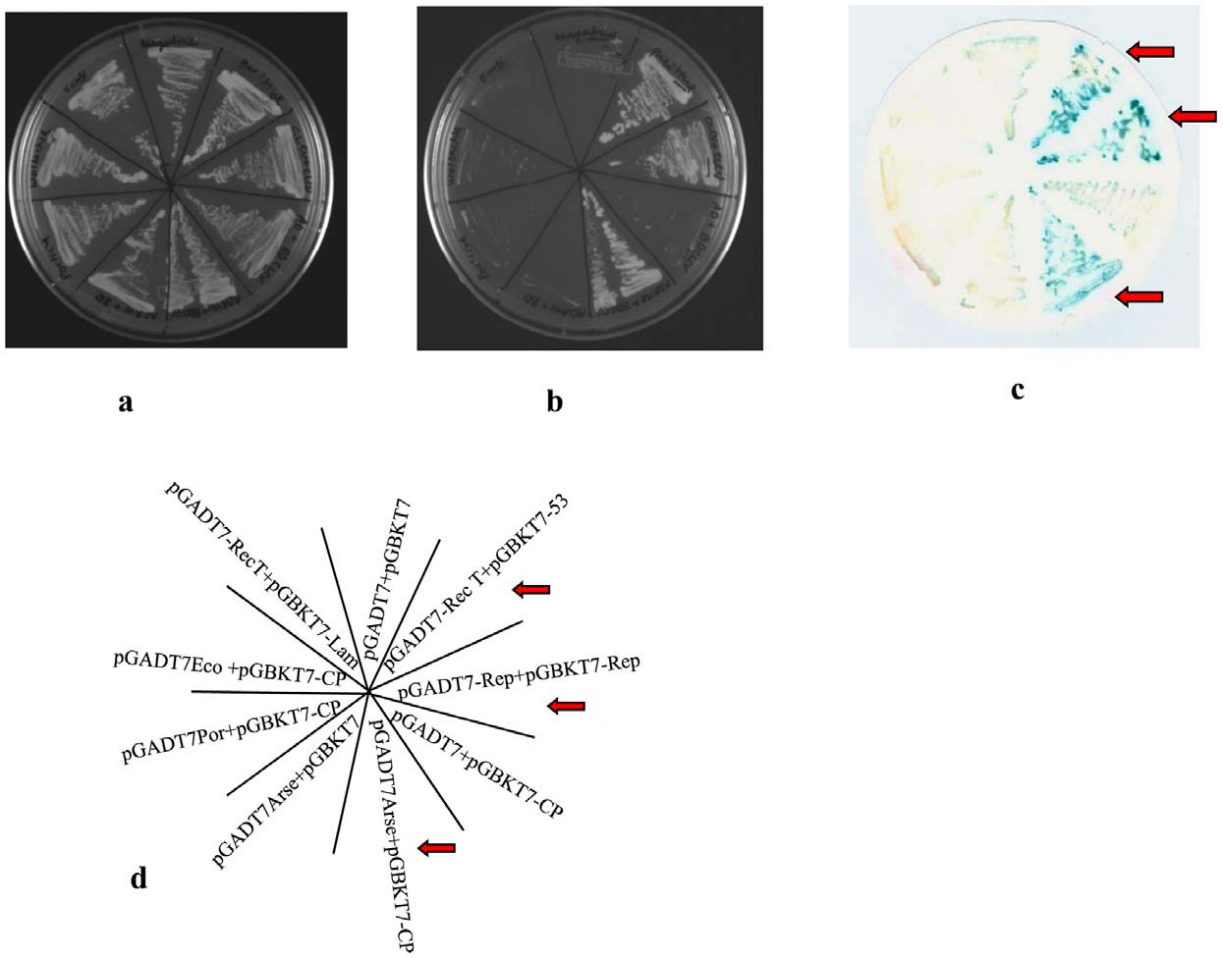
Yeast two hybrid analysis. Interaction of CLCuV coat protein with GroEL proteins obtained from *Arsenophonus*, *Portiera* and *E. coli* in yeast. Yeast cells were transformed with both plasmids constructs (AD and BD constructs) and all transformed cells were plated on 2 dropout media plate (**Figure 6.a.**). These cells were patched on 3 dropout media plates (**Figure 6.b.**). **β-gal assay of the cells grown in** **figure 6.b**. Positive controls and *Arsenophonus* GroEL-coat protein show β-gal activity and are indicated by red arrow (**Figure 6.c.**). Streaking pattern of constructs is indicated in **Figure 6.d.**.

Further interaction studies were performed using yeast two hybrid system in which the plasmid pGBKT- CP as well as either pGADT7-Arse GroEL or pGADT7-Por GroEL or pGADT7-Eco GroEL plasmid were co-transformed into yeast cells (AH109). pGADT7-Rep and pGBKT7-Rep [33] as well as one another set of plasmids pGADT7-T Ag (the SV40 large T-antigen fused to GAL4 DNA AD), and pGBKT7-53 (murine p53 fused to GAL4 DNA BD) provided with Kit were used as positive controls. pGBKT7-Lam+pGADT7-RecT and empty vectors were used as negative control. Yeast cells transformed with positive controls and pGADT7-Arse GroEL+pGBKT7-CP were able to grow on medium without histidine amino acid while no growth was observed with negative controls. pGADT7-Arse GroEL+pGBKT7 as well as pGADT7+pGBKT7-CP did not show any growth in Histidine lacking medium (Figure 6.b), ruling out the possibility of *de novo* activation of reporter gene in the presence of the expressed proteins. This indicated the specific interaction of *Arsenophonus* GroEL with CLCuV coat protein. *β-Galactosidase* expression occurred in yeast colonies harboring *Arsenophonus* GroEL+CLCuV coat protein, Rep-Rep, P53-T antigen (Figure 6.c), the last two being positive controls.

### *Arsenophonus* is present in salivary gland, midgut and bacteriocytes while *Portiera* was localized only in bacteriocytes

**Figure 7:**
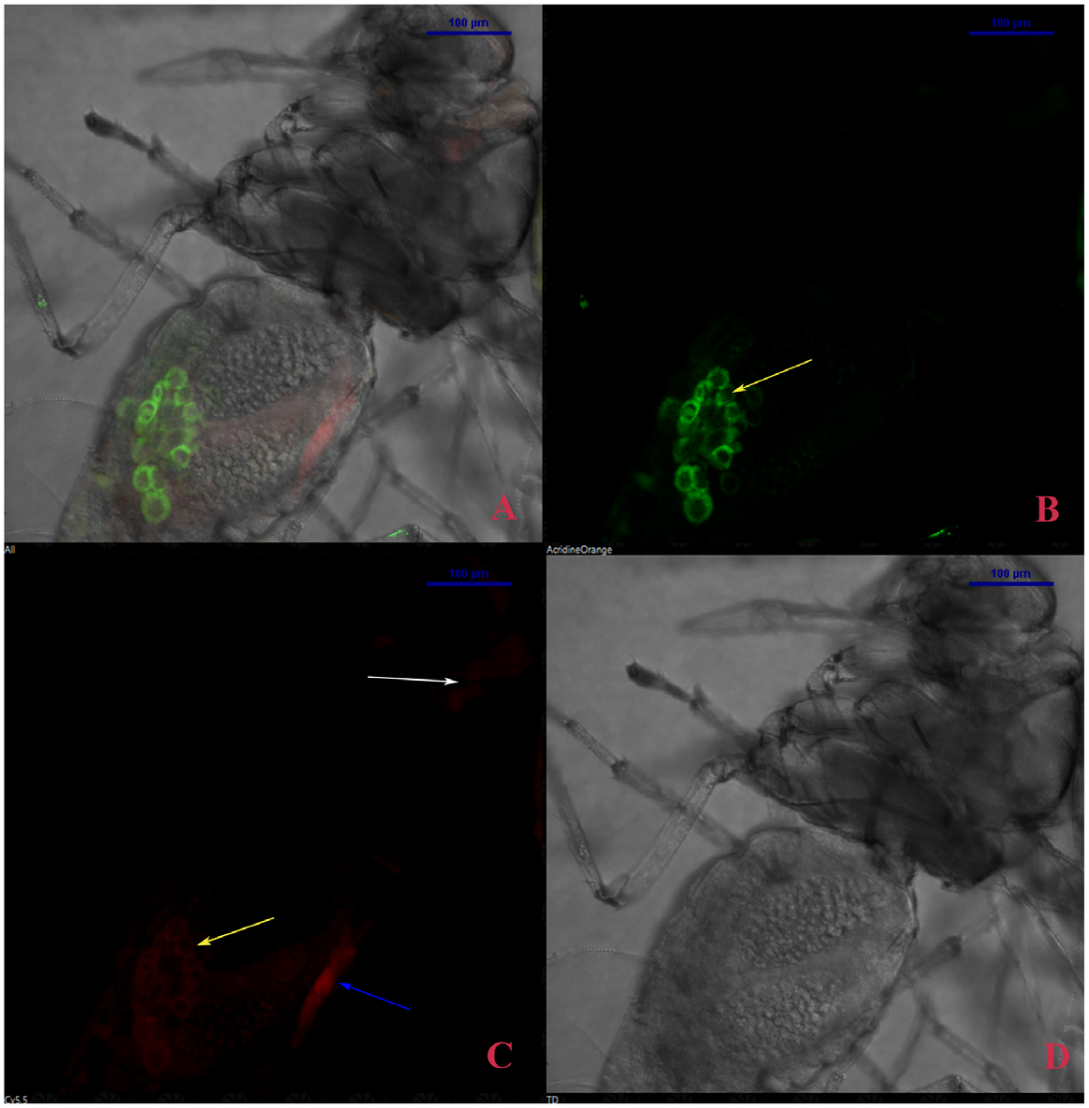
Localization of *Arsenophonus and Portiera* in *B. tabaci* adult female by FISH. *Arsenphonus* (red signal) was localized in the midgut, salivary gland and bacteriocytes of adult female *B. tabaci* (C) and *Portiera* (green signal) was absent in salivary gland as well as in midgut but present in the bacteriocytes (B). All images were viewed under 20× magnification. Arrows in yellow, white and blue indicate the bacteriocytes, salivary gland and midgut respectively. A and D panels show the merged and DIC images of the respective probe.

**Figure 8:**
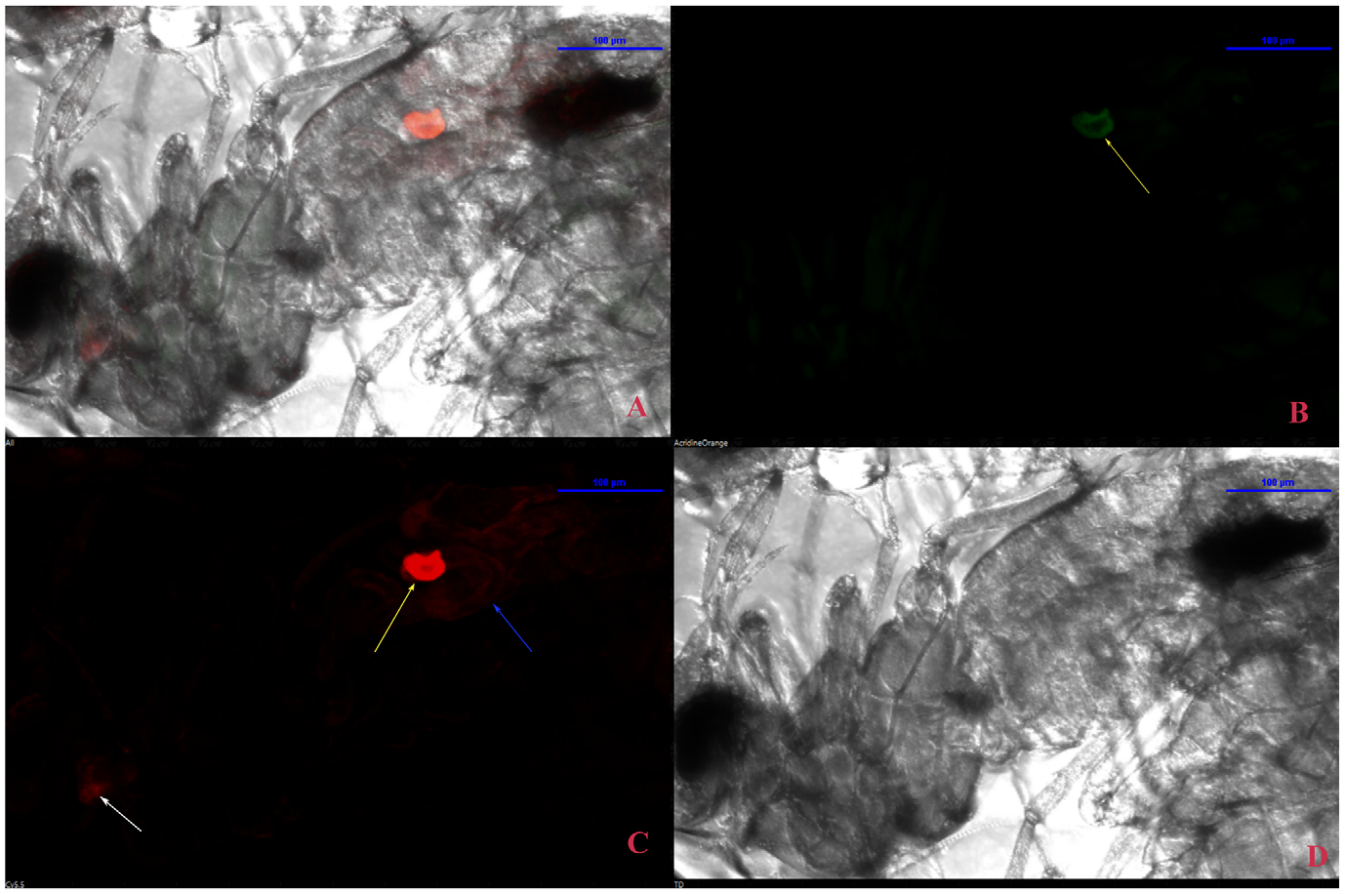
Localization of *Arsenophonus* and *Portiera* in *B. tabaci* adult male using FISH. *Arsenphonus* (red signal) was localized in the midgut, salivary gland and bacteriocytes of adult male *B. tabaci* (C) and *Portiera* (green signal) was absent in salivary gland as well as in midgut but present in the bacteriocytes (B). All images were viewed under 20× magnification. Arrows in yellow, white and blue indicate the bacteriocytes, salivary gland and midgut respectively. A and D panels show the merged and DIC images of the respective probe.

*Arsenophonus* and *Portiera* endosymbionts were localized with genus specific LNA probes containing TYE665 and FAM labeled respectively. The result indicated that *Arsenophonus* (red signal) was present in salivary glands, midgut and bacteriocytes of adult female flies (Figure 7.C) and male adult flies (Figure 8.C). Figure S4 shows the localization of *Arsenophonus* within the salivary glands, at a higher magnification. On the other hand *Portiera* (green signal), was present only in bacteriocytes while it was absent in salivary gland and midgut (Figure 7.B); (Figure 8.B). No probe flies and RNase digested flies were the negative control and they did not show any signals for these two endosymbionts. To check for bleed through or crossover of fluorescence signal (the emission of one flurophore detected by the filter combination of 2^nd^ flurophore), the flies were labeled separately with the two bacterial probes. No signal cross over was observed in these flies.

## Discussion

Cotton is an economically important kharif (monsoon season) cash crop of India which is being severely affected by CLCuD. In India, Sriganganagar area of Rajasthan has been the most affected region both by CLCuV and its insect vector [34]. *B. tabaci* is the primary vector transmitting Begomovirus to a large number of dicotyledonous plants. It has been reported that the Begomovirus acquired by *B. tabaci* can also be transmitted to the next generation through eggs [35]. TYLCCNV (*Tomato yellow leaf curl China virus*) can invade the ovary and fat bodies of *B. tabaci* and activate immune responses like autophagy. On the contrary, reports suggest that this virus also suppresses immune response by modulating the expression of genes related to Toll-like signaling and mitogen activated protein kinase pathways [36]. It is known that in *B. tabaci*, the ingested geminivirus particles are taken up in to the haemolymph via gut lumen where, it circulates and finally is secreted back into a fresh plant through salivary glands during feeding [37]. GroEL homologue protein, secreted by bacterial endosymbiont and *B. tabaci* protein BtHSP16 are the only identified proteins known to be involved in viral transmission pathway [26], [38]. Molecular details involved in the process of virus movement from gut to salivary glands remains largely unidentified. Geminivirus coat protein (CP) is the only known viral protein involved in virus transmission. Studies have shown that changes in amino acid sequences of coat protein can alter the vector specificity and transmission ability [39]. Exchanging the coat protein gene of *African cassava mosaic virus* (ACMV) with leafhopper-transmitted *Beet curly top virus* (BCTV) produces ACMV-BCTV CP chimera that can be transmitted by leafhopper [40]. Similarly coat protein replacement of nontransmissible AbMV with *Sida golden mosaic virus* produces a *B. tabaci* transmissible chimeric AbMV [41]

Recent studies have postulated that the *B. tabaci* midgut loop can exist in movable positions and it can move fully or partially in the thorax and abdomen. In a particular position the midgut loop makes direct contact with the salivary glands in the thorax region which could be the shortest route of virus transmission. But the direct evidence of virus transport is not yet available [42].

Bacterial endosymbionts make biotypes more invasive by providing them varying degree of virus transmission efficiency and sustainability in harsh environments like resistance to temperature and insecticides [32], [21]. In our study, we found that *B. tabaci* population of Sriganganagar was NonB biotype belonging to genetic group Asia II and the diversity of endosymbiotic bacterial population within these individuals was lower. Only *Arsenophonus* and *Portiera* were present whereas other previously reported endosymbionts of *B. tabaci* like *Hamiltonella* [17], *Rickettsia* [14], *Cardinium* and *Wolbachia* [43], [44] were not detected. This result suggested that only two endosymbionts, *Portiera* and *Arsenophonus* could be sufficient for maintaining *B. tabaci* fitness and efficient virus transmission. *Arsenophonus* was highly abundant suggesting its role in maintenance of the *B. tabaci* population as well as CLCuV infestation. The other endosymbiont *Portiera* is an obligate endosymbiont and is expected to maintain *B. tabaci* fitness by synthesizing essential amino acids [10]. Since, the primary endosymbiont *Portiera* is obligatory in nature; hence it has been reported in all *B. tabaci* biotypes like B, Q and NonB. This rules out its possibility of being involved in begomovirus transmission within its insect host.

It has been reported that the B biotype of *B. tabaci* has a good TYLCV transmission efficiency due to presence of *Hamiltonella* endosymbiont while, Q biotype is a poor transmitter because of the absence of same endosymbiont [24]. In contrast, Q biotype from Spain that harbors *Hamiltonella* has been shown to posses high virus transmission efficiency [24], [45].

But the fact that *Hamiltonella* is absent in our population inspite of the heavy field incidence of CLCuV disease prompted us to ask the question, whether any bacteria other than *Hamiltonella* could also interact with this virus? To assess the role of *Arsenophonus* and *Portiera* in CLCuV transmission, the GroEL proteins of both endosymbionts were purified and their interactions were checked with purified CLCuV coat protein. Results obtained by *in-vitro* pull down assay, co-immunoprecipitation and *in-vivo* yeast two hybrid analyses suggested that *Arsenophonus* GroEL interacts with CLCuV coat protein while, *Portiera* GroEL does not. In a similar study performed by Gottlieb *et al.* [24], with TYLCV coat protein, the GroEL protein of *Portiera* and *Arsenophonus* did not show any interaction. Although, we observed, some weak interaction of *E. coli* GroEL protein with CLCuV coat protein in *in-vitro* studies, there was no interaction in yeast two hybrid assay. *In- vitro* weak interaction of *E. coli* GroEL protein has also been reported in previous studies with PLRV (*Potato leafroll virus*) [46]. The disparity between our result with *Arsenophonus* GroEL and that by Gottlieb [24] could be attributed to the difference in begomovirus or the variation in GroEL protein of *Arsenophonus* isolated from Q biotype in Israel. Due to unavailability of information about the full coding sequence of *Arsenophonus* GroEL of Q Biotype of Israel [24], the variation among the *Arsenophonus* GroEL proteins could not be evaluated.

Interaction of endosymbiont GroEL protein with begomovirus particles seems to be an evolutionarily acquired adaptation shared by circulative transmitted plant viruses. Additionally, the comparative phylogenetic analysis of 16S rDNA and GroEL sequences of free living and endosymbiont bacteria also suggests that insect vectors have interacted with endosymbionts for at least last 100 million years [47]. GroEL proteins are chaperonin proteins which bind and stabilize the newly translated aggregation prone polypeptides [48]. They also mediate refolding process and assembly in an ATP-dependent manner [49], [50]. Unlike *E. coli* GroEL protein, *Buchnera* GroEL is not restricted to cytosol of bacteria so it can exist extracellularly in haemolymph [51], [52]. Similarly, in *B. tabaci* endosymbiont GroEL has been immunolocalized in haemolymph using antibody specific to *Buchnera* GroEL [26].

The interaction between endosymbiont GroEL and virus coat protein was first described in aphid transmitting luteovirus; barley yellow dwarf virus (BYDV) and the polerovirus potato leafroll virus (PLRV) [51], [23]. It was also shown that GroEL of aphid endosymbiont *Buchnera aphidicola* is not restricted to the bacteriocytes, but is also present in the haemolymph and gut [51], [52]. However, recent studies using Monoclonal antibody (MAb) raised against *Buchnera* specific GroEL epitopes, reveal that *Buchnera* GroEL is not present in organs, other than the bacteriocytes, like haemolymph, gut and fat body, questioning the possible role played by *Buchnera* GroEL as a receptor to the luteovirus coat protein [27]. Another recent study on aphid-leutovirus interaction disproved the earlier hypothesis that the read through domain (RTD) of the virus is essential for persistent and circulative transmission by the aphid [53].

Since GroEL protein binds to protein of wide range structures and sizes, including intermediates at various stages, it is difficult to validate a common phenomenon for GroEL protein substrates [48], [49], [50]. Similarly, in virus - GroEL interaction it is not understood that how GroEL protein acts as a chaperon because the diameter of GroEL cavity is 4.5 Å which is too small for the entry of large sized (∼18–30 nm) virus particles [27], [50]. The mode of interaction of virus particle with GroEL protein under *in-vivo* conditions is not fully understood and hence further studies in this direction are required.

Hence *in-vitro* interaction studies of coat protein with receptor candidates like GroEL should be confirmed with *in-vivo* experiments on whole insects. Unfortunately, as all the bacterial endosymbionts are not culturable and amenable to modern genetic manipulation techniques, proving these hypotheses under *in-vivo* insect conditions seem technically challenging at the moment. Further we should also explore for natural variation in endosymbiont GroEL sequence in *Bemisia tabaci*, having varying ability to transmit viruses which could throw light on the actual role if any, of GroEL in the virus transmission pathway.

In the previous studies, *Arsenophonus* was localized only inside the bacteriocytes [14], [54], [55], whereas in *B. tabaci* samples collected from Sriganganagar, *Arsenophonus* was found to be localized also in the salivary gland and midgut of *B. tabaci*. This further strengthens the hypothesis that the GroEL protein of this endosymbiont might be involved in CLCuV transmission. In the previous studies, begomovirus was localized in salivary gland using immunolocalization and *in-situ* hybridization methods, suggesting that salivary glands are crucial for virus transmission [56], [57], [58], [42]. Hence the presence of *Arsenophonus* in the salivary glands indicates that the GroEL protein of this endosymbiont may be protecting the virus particles in the salivary glands from proteolytic enzymes, as they do in the haemolymph [24], [44], thus maintaining the required titer of the virus particles for further transmission.

## Conclusion

*Arsenophonus* GroEL shows specific interaction with CLCuV coat protein, however, *in-vivo* evidence of this interaction is lacking. Using LNA probes, *Arsenophonus* could be localized in organs like, midgut and salivary glands, (besides the bacteriocytes from where it has been previously described) which are important in the circulative transmission of begomovirus by *B. tabaci*. Results from this study suggest that *Hamiltonella* is not the only bacterial endosymbiont capable of interacting with geminivirus coat protein.

## Materials and Methods

### Whiteflies and virus source

All *B. tabaci* samples used in the present study were collected from cotton (*Gossypium hirsutum*) field (Sriganganagar, Rajasthan). Samples were collected in 95% Ethanol and stored in −70°C till further use. For localization of endosymbionts, samples were collected in acetone and stored in −20°C.

### DNA Isolation

Single fly was washed with sterile water and homogenized with 14 µl of lysis buffer (100 mM Tris-Cl pH 8.0, 100 mM NaCl, 100 mM EDTA pH 8.0, 1%SDS and 1% proteinase K). Homogenated sample was incubated at 65°C for 30 min. Further, 27 µl of prechilled solution containing 6 M lithium chloride and 5 M potassium acetate was added and incubated on ice for 15 min. Further samples were centrifuged at 10,000 rpm, 4°C for 15 min. The supernatant was transferred into fresh microfuge tube and DNA was precipitated by centrifuged 10,000 rpm, 4°C for 15 min after treatment of 0.6 volume of isopropanol. Obtained DNA Pellet was washed with 70% ethanol. The air dried pellet was dissolved in Tris buffer (10 mM Tris-Cl, pH 8.0) and treated with RNase at 37°C for 30 minutes and stored at −20°C for further use.

### Identification of *B.tabaci* Genetic group

The *mtco1* gene from individual *B. tabaci* were amplified using Forward Primer- C1-J-2195 (5′-TTGATTTTTTGGTCATCCAGAAGT-3′) and Reverse Primer- L2-N-3014 (5′-TCCAATGCACTAATCTGCCATATTA-3′) [59]. The 25 µl reaction mix consisted of dNTP (2.5 mM), 10× Taq buffers (2.5 µl), primers (7.5 pmoles) each, Taq polymerase (1 U), DNA template and water to make up the volume.

The reaction conditions were 94°C for 30 sec thereafter 35 cycles of 94°C – 30 sec, 50°C – 30 sec and 72°C- 1 min 30 sec.

A negative control containing no DNA template was kept with each reaction. The PCR product was eluted from the gel using Hi Yield Gel/PCR DNA mini kit (Real Biotech Corporation).

The purified PCR product was then cloned in sequencing vector pGEMT Easy (Promega).

Out of all the positive transformants obtained 3 white colonies were selected for plasmid isolation using Hi Yield Plasmid mini kit (Real Biotech Corporation). These plasmid samples were sequenced using the commercial sequencing facility of Macrogen, Korea. The resulting sequences were aligned with *mtco1* sequences used in figure 2 of Boykin *et al.* [9] to identify the genetic group to which our whitefly belonged.

### Identification of bacteria present

For identification of the endosymbiotic bacterial diversity of *B. tabaci*, 16S rRNA gene was amplified by using universal primers primers 27F and 1492R [60]. The 25 µl of PCR reaction contained 20 ng DNA template, 1 unit of Taq polymerase, 2.5 µl of 10× PCR buffer, 2.5 mM of DNTP mix, and 7.5 pmoles of each primer. DNA fragment was amplified using thermal cycler (Thermo Applied Biosystem, USA) with an initial denaturation at 94°C for 30 sec followed by 28cycles of 94°C for 30 sec, 55°C for 30 sec and 72°C for 1 min 30 sec.

For negative control, no DNA template was take in a reaction. Amplified PCR products were run on 0.8% Agarose gel and observed. The amplified DNA fragments were purified from agarose gel using Hi Yield Gel/PCR DNA mini elution kit (Real Biotech Corporation) and cloned into sequencing vector PGEMT Easy (Promega). Ligated product was transformed into *E. coli DH5alpha* strain. We got more than 100 positive clones per reaction. Out of these 50 colonies were inoculated in 5 ml LB broth (Himedia) at 37°C for over-night for plasmid isolation. Plasmids were isolated from overnight culture using Hi Yield Plasmid mini kit (Real Biotech Corporation). Purified plasmid samples were sent for sequencing to Macrogen, Korea. Both directional sequencing were performed using T7 and SP6 primers. Obtained sequences were stitched using Mac Vector (version 11.1.1) and compared with available known sequences on NCBI database using the BLAST algorithm and phylogenetic analysis.

### Cloning, Expression and purification of CLCuV coat protein

Coat protein (CP) gene of CLCuV was amplified with coat protein specific primer (Table 1) using *B. tabaci* genomic DNA as template. Amplified sequences were cloned into pGEMT Easy vector and confirmed by sequencing. The gene was recloned into pGEX-4T1 (Amersham Biosciences, USA) and pET28a vectors. These constructs were transformed into *E. coli* strain *(Rossetta gami 2 DE3 pLysS)*. Transformed cells were induced with 0.5 mM IPTG for 16 hours. Induced pellet was dissolved in Buffer A and lysed by sonication. The soluble fraction of lysate was incubated with GST-sepharose beads. The bound protein fractions were eluted with elution buffer (buffer A containing 10 mM reduced glutathione) and were dialyzed against buffer B (25 mM Tris-HCl [pH. 8.0], 2.5 mM EDTA, 75 mM NaCl, 2.5 mM DTT, 5.0 mM MgCl2, 25% glycerol). Dialyzed protein samples were stored in −20°C until further use. ). Western blotting was also performed to identify the proteins using anti- ToLCNDV coat protein rabbit polyclonal antibodies.

**Table 1:** Primers sequences used in study for genes isolation and cloning into expression vectors.

Name	Sequence(5′ 3′)	Tm
*Ecoli* GroEL gene forward*Ecoli* GroEL gene reverse	AGATCTATGGCAGCTAAAGACGTAAAATTC GTCGACTTACATCATGCCGCCC	55°C
*Portiera* GroEL gene forward*Portiera* GroEL gene reverse	GGATCCATGGCAGCAAAACAGATTAG GTCGACCTAAGATCTCATACCATTTAC CC	55°C
*Arsenophonous* GroEL gene forward*Arsenophonous* GroEL gene reverse	CCA TGG GCC A TC ATC ATC ATC ATC AC A TGG CAG CTA AAG AC CAG GTC GAC TTA CAT CAT ACC ATT CAT TCC	52°C
CLCuV CP gene forwardCLCuV CP gene reverse	CCG GAT CCA TGT CGA AGC GAG CTG C GAC GTC GAC TCA ATT CGT TAC AGA GTC	55°C

### Cloning, Expression and Purification of *E.coli, Portiera* and *Arsenophonus* GroEL proteins

GroEL genes were amplified using PCR with genus specific primers (Table 1) *Arsenophonus*, and *Portiera* GroEL genes were isolated from genomic DNA of *B. tabaci* collected from Sriganganagar. *E. coli (DH5 alpha)* genomic DNA was used as a template for PCR to isolate *E. coli* GroEL gene. Amplified products were cloned in pGEMT Easy vector (Promega, USA) and confirmed by sequencing. Genes were recloned into pET28a expression vector (Novagen) at their respective restriction enzyme sites (Details are given in Table 1). Constructs were confirmed by restriction digestion. Further all constructs were transformed into *E. coli BL-21* strain. Transformed cells were induced with 0.5 mM isopropyl-β-D-thiogalactopyranoside (IPTG) grown for 16 hours. Induced pellets were resuspended in buffer A (20 mM Tris-HCl [pH 8.0], 300 mM NaCl, 1 mM dithiothreitol [DTT], 10% glycerol, 0.5 mM phenylmethylsulfonyl fluoride, 0.1% Triton X-100, 1 µg of aprotinin/ml, 1 µg of pepstatin/ml, 1 µg of leupeptin/ml, and 1 mg of benzamidine/ml), and lysed by sonication. Lysate was centrifuged at high speed 12,000 rpm for 15 minute at 4°C. The soluble fraction of the lysate was incubated with Ni-nitrilotriacetic agarose beads (GE healthcare). The bound proteins fractions were eluted with elution buffer (buffer A containing 250 mM imidazole) and were dialyzed against buffer B (25 mM Tris-HCl [pH. 8.0], 2.5 mM EDTA, 75 mM NaCl, 2.5 mM DTT, 5.0 mM MgCl2, 25% glycerol). Further western blotting was performed to identify the proteins using anti-*E. coli* GroEL polyclonal rabbit antibody (sigma). The dialysed protein samples were stored at −20°C until further use.

### Pull down assay

Purified GST–CP fusion protein (5 µg) was incubated with equal amounts (5 µg) of purified His-tag GroEL proteins from all three endosymbiont bacteria (*Portiera, E. coli* and *Arsenophonus*) in binding buffer [25 mM Tris–HCl (pH 8.0), 75 mM NaCl, 2.5 mM EDTA, 2.5 mM DTT 5 mM MgCl2, and 1% NP-40] at 37°C for 30 min. In each complex, binding buffer equilibrated glutathione-S-sepharose beads (15 µl) was added. The mixture was incubated on shaker for 30 minutes at 37°C. The unbound protein fraction was separated from the resin by centrifugation at 4000 rpm for 3 min. The resin containing the bound protein was washed with 400 mM of NaCl in binding buffer. 30 µl of water and 10 µl of 4× sample buffer was added to resin and boiled for 5 minutes, thereafter, the samples were centrifuged briefly. Half the amount (20 µl) of supernatant was analysed by SDS PAGE. The protein bands were visualized by Coomassie blue staining.

### Immuno-precipitation

His tag GroEL protein and His tag coat protein were co-expressed. To achieve the co-expression of both proteins, CP gene was cloned into pET14b vector (Novagen) containing ampicillin selection marker while GroEL genes were already cloned in pET28a vector having kanamycin selection marker. Both constructs were co-transformed into *E. coli* expressing strain *(Rossetta gami 2 DE3 pLysS)*. Transformed cells were induced with 1 mM IPTG at 25°C for 16 hours. After analysis the co-expression of both proteins, lysates were prepared by sonication in PBS buffer. One milliliter of cell lysate (500 µg/ml protein) was incubated with 1 µg of anti-coat protein polyclonal antibody (antibody produced against the coat protein of ToLCNDV in rabbit); (a gift from Dr. V.G Malti, Principal Scientist, IARI) at 4°C for 1 hour. Equlibrated “protein-A agarose” beads (GE healthcare) were added to each lysate - antibody mixture, further the reactions were allowed to rock slowly at 4°C for 1 hour. The beads were pelleted at 2500 rpm for 10 minutes and extensively washed with 400 mM NaCl containing PBS buffer twice. The protein in the beads was eluted by boiling in SDS gel sample buffer. The eluted proteins were separated by SDS-PAGE and immunoblotted onto a nitrocellulose membrane (Hybond-C, Amersham Biosciences). Protein bands were visualized by first incubating with anti-His tag primary antibody (produced in mouse); (Sigma) followed by developing with alkaline phosphatase-conjugated anti-mouse secondary antibody (produced in goat); (Bangalore genei). The blot was developed by using NBT- BCIP as substrate.

### Yeast two hybrid

The yeast strain *AH109* and plasmids pGBKT7and pGADT7 were used from Matchmaker Two-Hybrid system 3 (Clontech Laboratories Inc., Mountain View, CA, USA).This system contained HIS3, ADE2, MEL1 and LacZ reporters which provides high-stringency assays. CP gene was cloned into pGBKT7 vector. Fwd primer 5′ CCGGATCCAAATGTCGAAGCGAGCTG C3′ and Rev primer 5′GACGTCGACTCAATTCGTTACAGAGTC 3′ were used to amplify the CP gene. Amplified CP gene was cloned into pGBKT7 vector at *BamHI* and *SalI* sites. All the three GroEL genes were cloned into pGADT7 vector at *EcoR1* and *Sal1* restriction sites. All the constructs were verified by restriction digestion and sequencing. The yeast two-hybrid assay was performed according to the Clontech Yeast Protocol Handbook (protocol No. PT3024-1.Version No. PR13103), Clontech) using reporter.

Appropriate combinations of pGADT7 and pGBKT7 constructs were co-transformed into Yeast strain *AH109*.Transformed cells were grown on Single dropout plates in the absence of Trp and Leu for selecting co-transformants.

β-galactosidase activity of streaked colonies was tested by using filter lift assay. Colonies were transfered on Whatman filter paper by placing it on the surface of streaked colonies. The filter paper was lifted carefully and submerged into liquid nitrogen for 10 second and allowed to thaw at room temperature. This freeze/thaw step was repeated 3–5 times, after which this filter paper was carefully placed on another filter paper presoaked in 5 ml buffer Z [0.1 M Na-phosphate buffer (pH 7.0), 0.001 M MgSO4, 0.01 M KCl and 20 ml 20% X-gal and 8 ml b-mercaptoethanol]. The filter paper was incubated at 30°C in dark. The development of blue colour was checked after 6–8 h.

### Confocal analysis

The *B. tabaci* specimens were processed using standardized method of Gottlieb *et al.* [14] with slight modifications. *B. tabaci* adult female specimens were stored overnight in Carnoy's fixative (chloroform∶ethanol∶glacial acetic acid, 6∶3∶1) and decolorized with 6% H_2_O_2_ in ethanol for 24 hrs. FAM labeled and TYE-665 labeled LNA probes bearing sequences of 5′ TGTCAGTGTCAGCCCAGAAG 3′ for *Portiera* and 5′ TCATGACCACAACCTCCAAA3 for *Arsenophonus* respectively (Gottleib *et al.*, 2008) were supplied by Exiqon A/S. The decolorized insects were hybridized at 40°C, with LNA probes for *Portiera* and *Arsenophonus*, in hybridization buffer (20 mM Tris-Cl [pH 8.0], 0.9 M NaCl, 0.01% sodium dodecyl sulfate) containing 50% formamide. Probe concentration was 0.6 pmoles. After the overnight incubation, the samples were thoroughly washed in a washing buffer (0.3 M NaCl, 0.03 M sodium citrate, 0.01% sodium dodecyl sulfate) for 5 minutes and mounted using Vectasheild (Vector Labs). Replicates consisted of 10 insects. All the images were acquired with Nikon A1 confocal microscope and analysed by NIS elements (V 3.21.02) image analysis software (Nikon).

## Supporting Information

Figure S1 **Heterologous expression and purification of 30 kDa CLCuV coat protein tagged with 23 kDa GST.** The GST tagged CLCuV coat protein was overexpressed in *E. coli Rossetta gami 2 DE3 PlysS* with 0.5 mM IPTG induction. Protein samples were resolved on 10% SDS- polyacrylamide gel. **Figure 1.a.** **Comparison of total proteins in uninduced and induced cells. Lane 1**-Molecular weight marker, **Lane 2**-total protein in uninduced cells, **Lane 3-** total protein in induced cell culture showing a prominent 52 kDa band of over expressed GST tagged CLCuV coat protein. **Figure 1.b.** **Purification of GST tagged CLCuV coat protein. Lane 1**- molecular weight marker. **Lane 2**- flowthrough obtained after the affinity binding of cell lysate with GST beads. **Lane 3**- wash fraction obtained after the GST beads bound with cell lysate was washed with buffer A containing 400 mM NaCl. **Lane 4-** the GST tagged CLCuV coat protein was eluted from GST bead using buffer A containing 10 mM glutathione. **Figure 1.c.**- **western blot analysis of purified protein using anti- coat protein antibody. Lane 1**- molecular weight marker. **Lane 2**- 52 kDa GST tagged CLCuV coat protein. **Lane 3**- bacterial cell lysate (without coat protein) to check nonspecific binding.
(TIF)

Figure S2 **Purification of** ***E.coli*** **,** ***Portiera*** **and** ***Arsenophonus*** **GroEL proteins.** **Figure 2.a.** **Purification of ∼66 kDa** ***E. coli*** **GroEL protein tagged with 0.6 kDa Hisidine tag. Lane 1**- molecular weight marker. **Lane 2**- flow through obtained after the affinity binding of the soluble fraction of induced bacterial lysate with Ni- NTA beads. **Lane 3**- **4**- wash fractions obtained after washing of beads with buffer A containing 10 mM and 50 mM immidazole respectively. **Lane 5–7-** eluted fractions of *E. coli* GroEL protein. **Figure 2.b.** **Purification of ∼66 kDa** ***Portiera*** **GroEL protein tagged with 0.6 kDa HIS. Lane 1**- molecular weight marker. **Lane 2**- flowthrough obtained after the affinity binding of the soluble fraction of induced bacterial lysate with Ni- NTA beads. **Lane 3 and Lane 4**- wash fractions obtained after washing of beads with buffer A containing 10 mM and 50 mM immidazole respecively. **Lanes 5–7** – eluted fractions of *Portiera* GroEL protein. **Figure 2.c.** **Purification of ∼66 kDa** ***Arsenophonus*** **GroEL protein tagged with 0.6 kDa HIS Lane 1**- Molecular weight marker. **Lane 2**- pellet fraction of induced bacterial lysate. **Lane 3**- flowthrough obtained after the affinity binding of the soluble fraction with Ni- NTA beads. **Lanes 4 and 5-** wash fractions obtained after washing of beads with buffer A containing 10 mM and 50 mM immidazole respecively. **Lanes 6–10**- eluted fractions of *Arsenophonus* GroEL protein.
(TIF)

Figure S3 **Western blot analysis of purified GroEL proteins.** Confirmation of *Arsenophonus*, *E. coli* and *Portiera* GroEL proteins by western blotting with anti *E. coli* GroEL antibody. **Figure 3.a.** **Purified GroEL proteins obtained from** ***Arsenophonus*** **,** ***E. coli*** **, and** ***Portiera*** **on SDS PAGE stained with coomassie brilliant blue dye Lane 1-** molecular weight marker. **Lane 2-** *Arsenophonus* GroEL, **Lane 3-** *E. coli* GroEL, **Lane 4-** *Portiera* GroEL. **Figure 3.b.** – purified GroEL proteins obtained from *Arsenophonus*, *E. coli*, and *Portiera* were immunoblotted with anti *E. coli* GroEL antibody and bands were visualized using NBT-BCIP substrate. **Lane 1-** molecular weight marker. **Lane 2**- *Arsenophonus* GroEL **Lane 3**- *E. coli* GroEL. **Lane 4**- *Portiera* GroEL.
(TIF)

Figure S4 **Localization of** ***Arsenophonus*** **in salivary gland of adult** ***B. tabaci*** **at 40× magnification.** *Arsenophonus* (red signal) was detected in salivary gland (C) while *Portiera* (green signal) was completely absent (B). A and D panels show the merged and DIC images of the respective probe. Arrow in white indicates the salivary gland.
(TIF)
