# Supplementary figures and images for: Arsenophonus GroEL Interacts with CLCuV and Is Localized in Midgut and Salivary Gland of Whitefly B. tabaci
Source: PLoS One. 2012 Aug 10;7(8):e42168. doi: 10.1371/journal.pone.0042168 (PMC3416813; doi:10.1371/journal.pone.0042168)

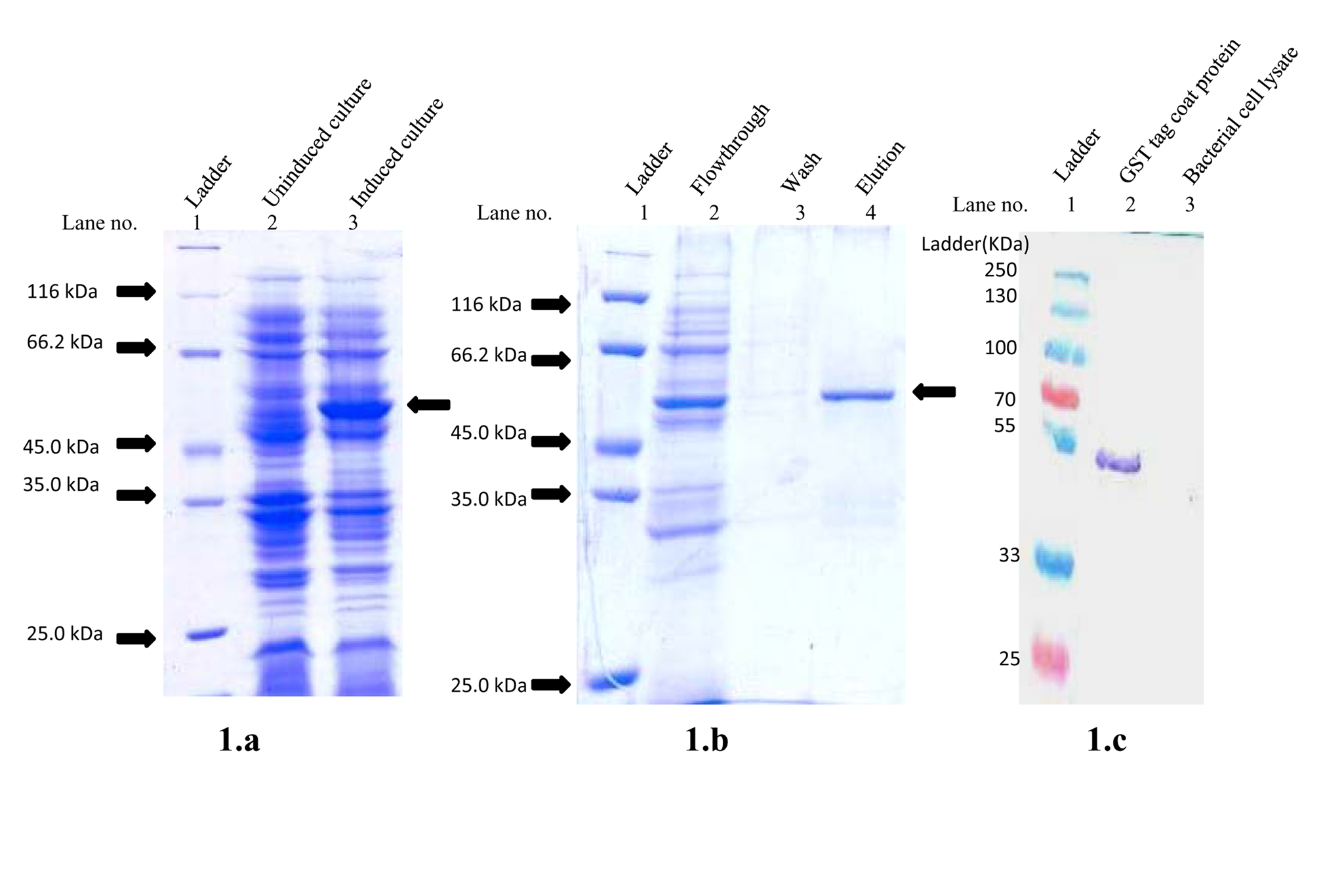

Supplement: Figure S1 — Heterologous expression and purification of 30 kDa CLCuV coat protein tagged with 23 kDa GST. The GST tagged CLCuV coat protein was overexpressed in E. coli Rossetta gami 2 DE3 PlysS with 0.5 mM IPTG induction. Protein samples were resolved on 10% SDS- polyacrylamide gel. Figure 1.a. Comparison of total proteins in uninduced and induced cells. Lane 1-Molecular weight marker, Lane 2-total protein in uninduced cells, Lane 3- total protein in induced cell culture showing a prominent 52 kDa band of over expressed GST tagged CLCuV coat protein. Figure 1.b. Purification of GST tagged CLCuV coat protein. Lane 1- molecular weight marker. Lane 2- flowthrough obtained after the affinity binding of cell lysate with GST beads. Lane 3- wash fraction obtained after the GST beads bound with cell lysate was washed with buffer A containing 400 mM NaCl. Lane 4- the GST tagged CLCuV coat protein was eluted from GST bead using buffer A containing 10 mM glutathione. Figure 1.c.- western blot analysis of purified protein using anti- coat protein antibody. Lane 1- molecular weight marker. Lane 2- 52 kDa GST tagged CLCuV coat protein. Lane 3- bacterial cell lysate (without coat protein) to check nonspecific binding. (TIF) [file pone.0042168.s001.tif]

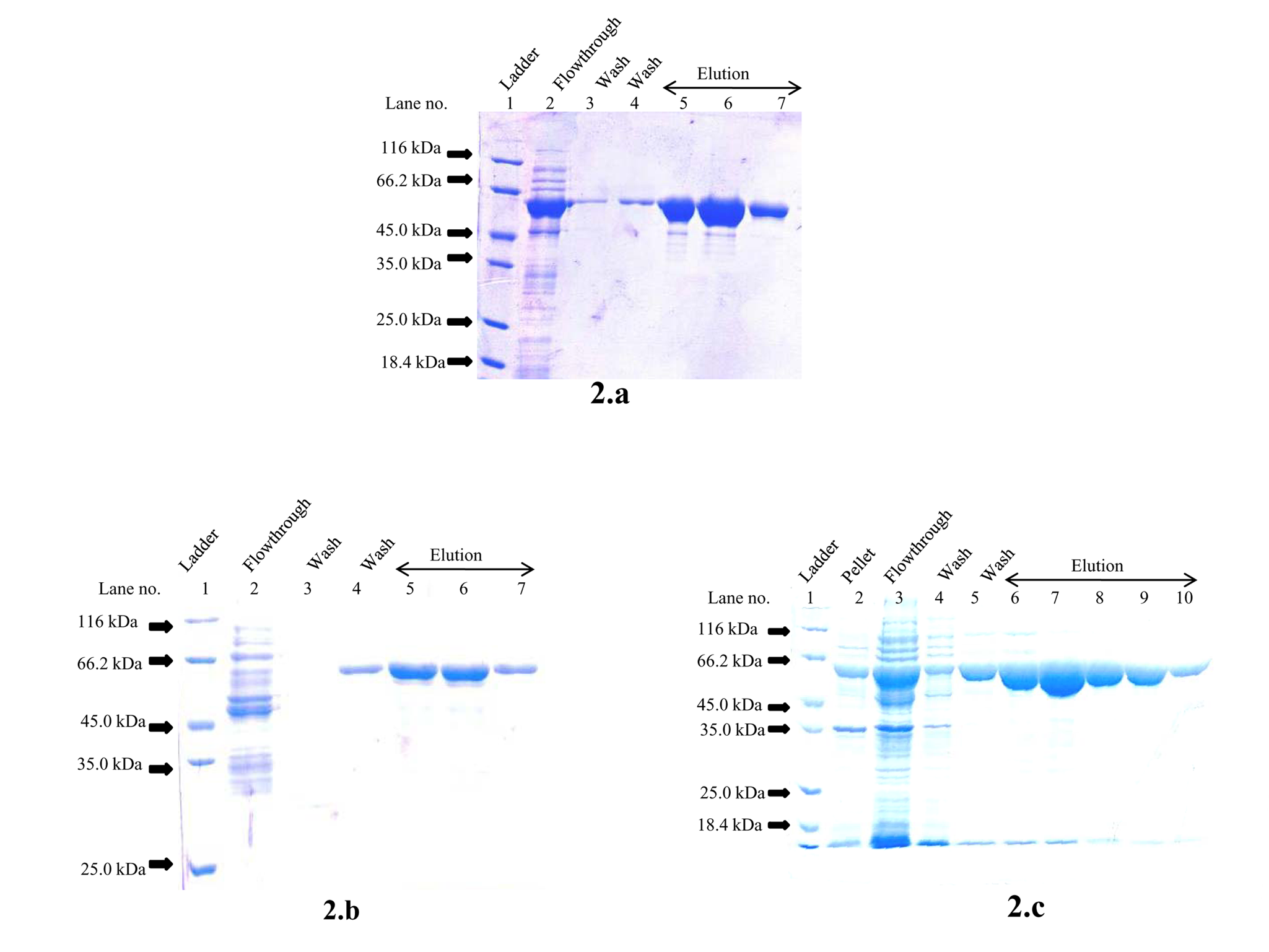

Supplement: Figure S2 — Purification of E.coli , Portiera and Arsenophonus GroEL proteins. Figure 2.a. Purification of ∼66 kDa E. coli GroEL protein tagged with 0.6 kDa Hisidine tag. Lane 1- molecular weight marker. Lane 2- flow through obtained after the affinity binding of the soluble fraction of induced bacterial lysate with Ni- NTA beads. Lane 3- 4- wash fractions obtained after washing of beads with buffer A containing 10 mM and 50 mM immidazole respectively. Lane 5–7- eluted fractions of E. coli GroEL protein. Figure 2.b. Purification of ∼66 kDa Portiera GroEL protein tagged with 0.6 kDa HIS. Lane 1- molecular weight marker. Lane 2- flowthrough obtained after the affinity binding of the soluble fraction of induced bacterial lysate with Ni- NTA beads. Lane 3 and Lane 4- wash fractions obtained after washing of beads with buffer A containing 10 mM and 50 mM immidazole respecively. Lanes 5–7 – eluted fractions of Portiera GroEL protein. Figure 2.c. Purification of ∼66 kDa Arsenophonus GroEL protein tagged with 0.6 kDa HIS Lane 1- Molecular weight marker. Lane 2- pellet fraction of induced bacterial lysate. Lane 3- flowthrough obtained after the affinity binding of the soluble fraction with Ni- NTA beads. Lanes 4 and 5- wash fractions obtained after washing of beads with buffer A containing 10 mM and 50 mM immidazole respecively. Lanes 6–10- eluted fractions of Arsenophonus GroEL protein. (TIF) [file pone.0042168.s002.tif]

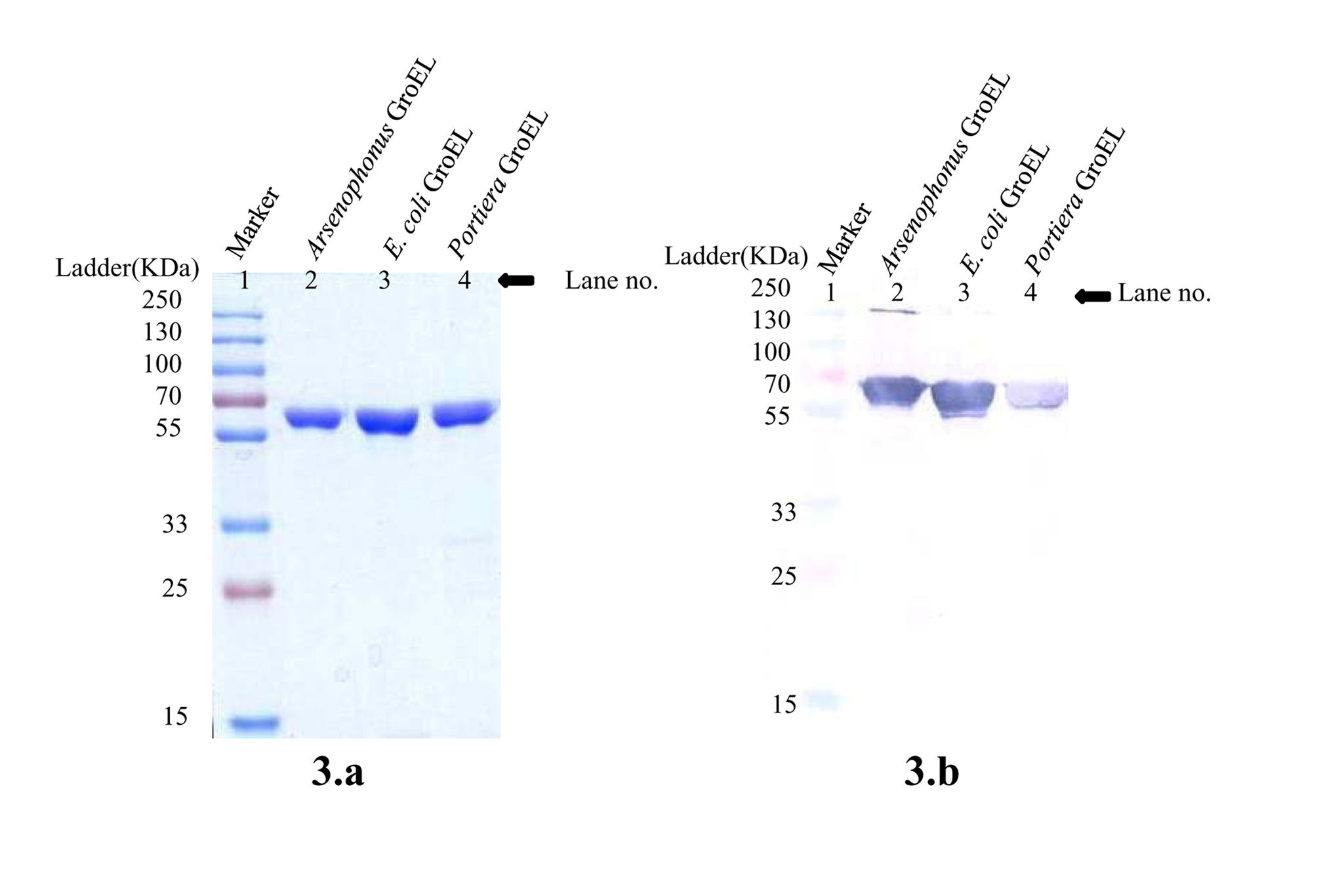

Supplement: Figure S3 — Western blot analysis of purified GroEL proteins. Confirmation of Arsenophonus, E. coli and Portiera GroEL proteins by western blotting with anti E. coli GroEL antibody. Figure 3.a. Purified GroEL proteins obtained from Arsenophonus , E. coli , and Portiera on SDS PAGE stained with coomassie brilliant blue dye Lane 1- molecular weight marker. Lane 2- Arsenophonus GroEL, Lane 3- E. coli GroEL, Lane 4- Portiera GroEL. Figure 3.b. – purified GroEL proteins obtained from Arsenophonus, E. coli, and Portiera were immunoblotted with anti E. coli GroEL antibody and bands were visualized using NBT-BCIP substrate. Lane 1- molecular weight marker. Lane 2- Arsenophonus GroEL Lane 3- E. coli GroEL. Lane 4- Portiera GroEL. (TIF) [file pone.0042168.s003.tif]

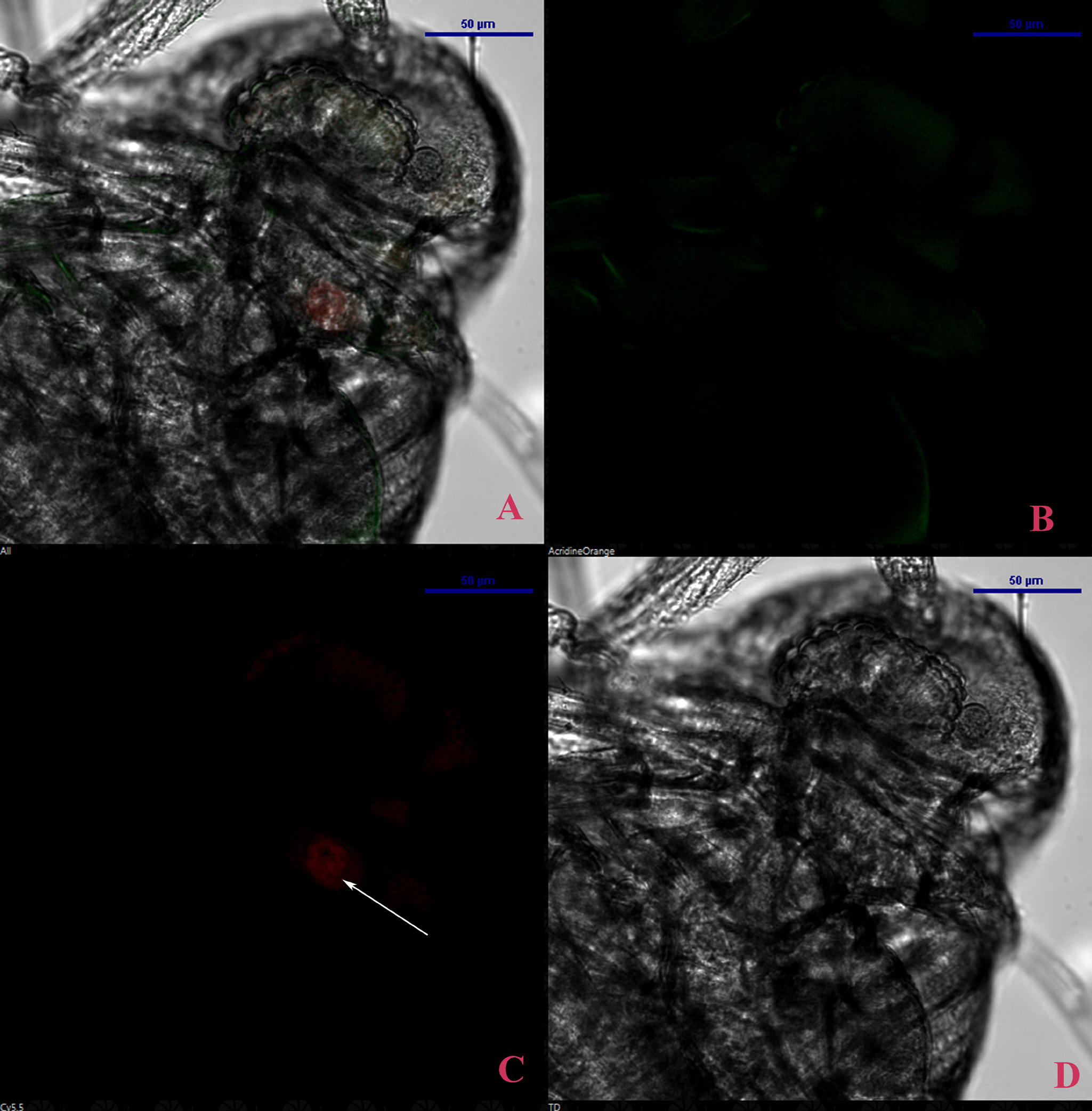

Supplement: Figure S4 — Localization of Arsenophonus in salivary gland of adult B. tabaci at 40× magnification. Arsenophonus (red signal) was detected in salivary gland (C) while Portiera (green signal) was completely absent (B). A and D panels show the merged and DIC images of the respective probe. Arrow in white indicates the salivary gland. (TIF) [file pone.0042168.s004.tif]
